# Exposing the Causal Effect of Body Mass Index on the Risk of Type 2 Diabetes Mellitus: A Mendelian Randomization Study

**DOI:** 10.3389/fgene.2019.00094

**Published:** 2019-02-14

**Authors:** Liang Cheng, He Zhuang, Hong Ju, Shuo Yang, Junwei Han, Renjie Tan, Yang Hu

**Affiliations:** ^1^College of Bioinformatics Science and Technology, Harbin Medical University, Harbin, China; ^2^Department of Information Engineering, Heilongjiang Biological Science and Technology Career Academy, Harbin, China; ^3^School of Life Sciences and Technology, Harbin Institute of Technology, Harbin, China

**Keywords:** body mass index, type 2 diabetes mellitus, casual effect, Mendelian randomisation, phenotype

## Abstract

**Introduction:** High body mass index (BMI) is a positive associated phenotype of type 2 diabetes mellitus (T2DM). Abundant studies have observed this from a clinical perspective. Since the rapid increase in a large number of genetic variants from the genome-wide association studies (GWAS), common SNPs of BMI and T2DM were identified as the genetic basis for understanding their associations. Currently, their causality is beginning to blur.

**Materials and Methods:** To classify it, a Mendelian randomisation (MR), using genetic instrumental variables (IVs) to explore the causality of intermediate phenotype and disease, was utilized here to test the effect of BMI on the risk of T2DM. In this article, MR was carried out on GWAS data using 52 independent BMI SNPs as IVs. The pooled odds ratio (OR) of these SNPs was calculated using inverse-variance weighted method for the assessment of *5 kg/m^2^* higher BMI on the risk of T2DM. The leave-one-out validation was conducted to identify the effect of individual SNPs. MR-Egger regression was utilized to detect potential pleiotropic bias of variants.

**Results:** We obtained the high OR (1.470; 95% CI 1.170 to 1.847; *P* = 0.001), low intercept (0.004, *P* = 0.661), and small fluctuation of ORs {from -0.039 [(1.412 – 1.470) / 1.470)] to 0.075 [(1.568– 1.470) / 1.470)] in leave-one-out validation.

**Conclusion:** We validate the causal effect of high BMI on the risk of T2DM. The low intercept shows no pleiotropic bias of IVs. The small alterations of ORs activated by removing individual SNPs showed no single SNP drives our estimate.

## Introduction

Diabetes mellitus (DM) is characterized by a bunch of chronic metabolic diseases leading to insulin-secretion deficiency (Olokoba et al., 2012; Pan et al., 2013; Shi and Hu, 2014). High blood sugar levels in DM patients over a prolonged period impair body tissues, such as eye, kidney, heart, and so on. Currently, more than 400 million people suffer from diabetes worldwide, of which type 2 DM (T2DM) makes up about 90% (Olokoba et al., 2012; Pan et al., 2013; Shi and Hu, 2014). Most patients who suffer from T2DM are over the age of 40 (Olokoba et al., 2012; Pan et al., 2013; Shi and Hu, 2014). In theory, people have a long time to prevent T2DM under the right direction. To this end, researchers go out of their way to investigate the causes of T2DM.

Observational studies exposed that body mass index (BMI) was strongly associated with the risk of being diagnosed with T2DM (Sanada et al., 2012; Ganz et al., 2014; Chen et al., 2015, 2016; Zhao et al., 2017). In Sanada et al. (2012) conducted a 10-year retrospective cohort study on 969 men and 585 women (Sanada et al., 2012). They observed high BMI was an independent and dose-dependent risk factor for T2DM in Japanese patients (Sanada et al., 2012). In Ganz et al. (2014) directed a case-control study to assess the association between BMI and the risk of T2DM in the United States (Ganz et al., 2014). A positive association between them was found in 12,179 cases (> = 18 years old) and 25,177 controls (Ganz et al., 2014). The analogous studies without considering genetic factors almost came to a consistent conclusion.

After identifying a large number of BMI-associated and T2DM-associated loci in genome-wide association studies (GWAS), their common associated variants were then interpreted as the underlying cause of BMI and the risk of T2DM. In 2007, the first common variant in the FTO gene of BMI and T2DM was reported in European descents (Frayling et al., 2007). Subsequently, corresponding investigations sprung up for validating the existing common locus and identifying their novel common variants of BMI and T2DM (Andreasen et al., 2008; Herder et al., 2008; Cauchi et al., 2009; Legry et al., 2009; Webster et al., 2010; Song et al., 2012; Xi et al., 2014). In 2014, a meta-analysis of 42 studies for BMI and T2DM associated variants was conducted (Xi et al., 2014). Eventually, 4 statistically significant associated variants (FTO rs9939609, SH2B1 rs7498665, FAIM2 rs7138803, GNPDA2 rs10938397) were identified for both in Europeans.

**FIGURE 1:**
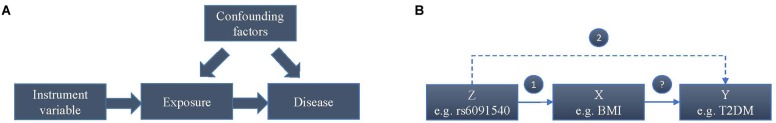
Mendelian randomisation analysis using genetic variants as instrumental variables for estimating the influence of BMI on T2DM. **(A)** Causal effect in Mendelian randomisation. **(B)** The basic principle of estimating the influence of BMI on the risk of T2DM.

Whether a higher BMI increases the risk of T2DM or T2DM affects BMI or their common genetic factors take effect, is still unknown according to current observations. In addition, after considering confounding factors, the causal relationship between BMI and T2DM may be reverse. To estimate the causal effect of BMI on the risk of T2DM, we conducted this Mendelian randomization (MR) study, which is an instrumental variable (IV) based method to infer causality of exposure and disease in observation studies. Genetic variants that are associated with intermediate phenotypic exposures are introduced as IVs by MR to estimate the effect of phenotypic exposures on a disease outcome (Figure 1A). Due to random distribution of gene variants during gametogenesis, IV-based analysis can avoid reverse causality. The basic principle of estimating the influence of BMI on the risk of T2DM using MR is shown in the Figure 1B, where Z (e.g., variants) represents IV, X indicates exposure BMI, and Y is disease T2DM. Two assumptions should be suitable for the case before using MR.

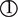
 The variants are robustly associated with BMI.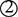 The variants are independent of the T2DM without considering BMI and confounders. It means the only way to influence the T2DM by the variants is via an intermediate.

The two assumptions mean the variants should be associated with BMI but not with T2DM. Therefore, the conclusions based on MR could not result from the common genetic factors of BMI and T2DM.

## Materials and Methods

Two summary-level data of GWAS datasets were utilized by MR analysis. One of them was for extracting significant BMI SNP sets to meet the assumption 1. And the other was for extracting no significant T2DM SNP sets to meet assumption 2. The intersections of these two SNP sets were then analyzed using MR.

### Summary-Level Data for Associations Between Genetic Variants and BMI

In Locke et al. (2015) conducted a meta-analysis of BMI using GWAS on Metabochip studies (Voight et al., 2012). Totally, 322,154 individuals of European descents and 17,072 individuals of non-European descent were analyzed. As a result, 97 BMI-associated SNPs (*P* < 5 × 10^-8^) were identified for European. The corresponding SNPs, effect allele (EA), allele frequencies, beta coefficients, and standard errors (SEs) were extracted from Genetic Investigation of Anthropometric Traits (GIANT) consortium (Locke et al., 2015) as summary-level data for associations between genetic variants and BMI.

### Summary-Level Data for Associations Between Genetic Variants and T2DM

Morris et al. (2012) carried out a combined meta-analysis of European descents on two GWAS data sets (Yang et al., 2010; Lee et al., 2011), which involved 22,669 cases and 58,119 controls. All the variants were then genotyped with Metabochip involving 1,178 cases and 2,472 controls of Pakistani descent. The analytical result contains novel susceptibility locus together with other SNPs, SEs and their *P*-values on the risk of T2DM. These were utilized as summary-level data for associations between genetic variants and T2DM.

### Data Processing and Analysis

**FIGURE 2:**
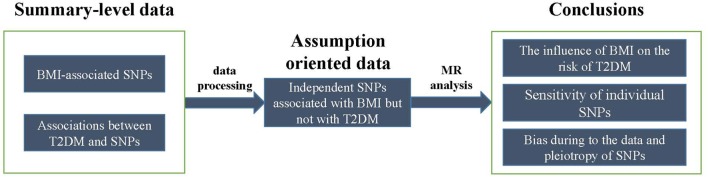
The schematic of data processing and analysis.

Two summary-level datasets were processed into assumption-oriented data (Figure 2). According to assumption 2, genetic pleiotropy can result in over-precise estimates in subsequent analysis. According to the application principles of Mendelian randomization analysis, the study is based on Mendel’s second law of inheritance: the separation and combination of genetic gametes controlling different traits do not interfere with each other; in the formation of gametes, the paired genetic gametes that determine the same trait are separated from each other, and the genetic gametes that determine different traits are freely combined. When the two genes are not completely independent, they will show a certain degree of linkage, a situation called linkage disequilibrium (LD), which will greatly affect the exclusiveness of the variable tool to phenotypic inheritance, leading the subsequent calculations bias generally called “over-precise estimates.” To avoid this situation, these loci with potential LD were removed from 97 BMI-associated SNPs, which was done by Noyce et al. (2017) in the previous study. The 97 SNPs were first ranked from the smallest to largest *P*-values. Then for the top ranked SNPs, Noyce et al. (2017) removed those in LD (*R*^2^ threshold of 0.001) or those within 10,000 kb physical distance based on a reference dataset (Devuyst, 2015) from the 97 SNPs. This process was iterated for the remaining SNPs. As a result, 78 BMI-associated SNPs (*P* < 5 × 10^-8^) without potential LD of each other were obtained. According Xi et al. (2014), meta-analysis, four SNPs (rs9939609, rs7498665, rs7138803, rs10938397) were found at the T2DM-associated locus, and were also further removed from these 78 SNPs. In addition, those SNPs with *P*-value less than 0.05 by Morris et al. (2012) were removed as well. Finally, 52 SNPs that confirmed to the two MR assumptions were retained for MR analysis.

Three subjects involving the influence of BMI on the risk of T2DM (Figure 2), the sensitivity of the disproportionate effects of variants, and the detection of bias due to pleiotropy were investigated in MR analysis. These issues were analyzed by MR method, leave-one-out validation, and MR-Egger regression (Bowden et al., 2015), respectively.

• MR method

MR method was described in the previous study (Bowden et al., 2015) and summarized for evaluating the influence of BMI on the risk of T2DM as below. Assuming *X, Y*, and *Z* are BMI, T2DM, and variants, respectively, Wald ratio (*β_XY_*) of BMI to T2DM through specified variant is calculated as follows:

(1) $$\beta_{XY}=\beta_{ZY}/\beta_{ZX},$$

where *β_ZY_* represents the per-allele *log(OR)* of T2DM from summary-level data of Morris et al. (2012) study. *β_ZX_* is the per-allele *log(OR)* of BMI from summary-level data of Locke et al. (2015) study. SE of BMI-T2DM association of each Wald ratio is defined as follows:

(2) $${SE}_{XY}={SE}_{ZY}/{SE}_{ZX},$$

where *SE_ZY_* and *SE_ZX_* represent the *SE* of the variant-T2DM and variant-BMI associations from corresponding summary-level data, respectively. Subsequently, 95% confidence intervals (CIs) were then calculated from the *SE* of each Wald ratio. These summarized data were then estimated using inverse-variance weighted (IVW) linear regression for meta-analysis. The meta-analysis model for the point estimate is according to the heterogeneity of the summarized data. Fixed effect model is used for no significant heterogeneity, and random-effect model is used for others.

To evaluate the genetic heterogeneity of summarized data, Cochran’s *Q*-test and statistic *I^2^* were utilized here. Cochran’s *Q*-test follows a *χ*^2^ distribution with *k-1* degrees of freedom, where k represents the number of variants for analysis. *I^2^ = (Q-(k-1))/Q × 100%* ranges from 0 to 100%. *P < 0.01* and *I^2^ > 50%* were defined as the significant heterogeneity here (Zhang et al., 2015).

• Leave-one-out validation

To test the sensitivity of variants, we designed a leave-one-out validation measure. In brief, to test the influence of an SNP to the conclusion, the SNP was removed from the 52 SNPs to carry out IVW point estimate. The fluctuation of the results before and after removing the SNP reflects the sensitivity of this SNP. Here this process was iterated for each of these 52 SNPs.

• MR-Egger test

To ensure that violations of our analysis were not biasing the estimate of the directional causal association, MR-Egger regression asymmetry test was used (Bowden et al., 2015). The MR-Egger regression is adapted from Egger regression, which is a tool to detect small study bias in meta-analysis and test for bias from pleiotropy. The estimated value of the intercept in MR-Egger regression can be interpreted as an estimate of the average pleiotropic effect across the genetic variants. An intercept that differs from zero is indicative of overall directional pleiotropy. The slope coefficient from MR-Egger regression provides a bias estimate of the causal effect.

All statistical tests for MR analysis were undertaken using the R Package of meta-analysis^1^ and Mendelian Randomization (Yavorska and Burgess, 2017).

## Results

Among the 97 BMI-associated SNPs (Locke et al., 2015), 19 SNPs with LDs, 2 T2DM-associated SNPs (rs7138803, rs10938397) from Xi et al. (2014) study, 20 T2DM-associated SNPs and 1 unmapped SNPs from Morris et al. (2012) study, and 3 uncertain SNPs were removed (Supplementary Table 1). 52 BMI-associated SNPs were eventually selected for the MR analysis in Table 1. Each line of the table documents 12 items involving the SNP, EA and its frequencies, beta coefficients of the SNP on the risk of BMI and T2DM, and SEs.

**Table 1:** Associations of genetic variants with BMI and T2DM.

				BMI			T2DM	T2DM	T2DM
SNP	Chr	Gene	BP	beta	BMI SE	BMI P	beta	SE	P
rs977747	1	TAL1(N) *TAL1*(N)	47457264	0.017	0.003	2.18E-08	-0.010	0.020	0.63
rs657452	1	AGBL4(N)	49362434	0.023	0.003	5.48E-13	-0.010	0.020	0.45
rs3101336	1	NEGR1(B,C,D,N)	72523773	0.032	0.003	2.66E-26	0.010	0.020	0.66
rs12401738	1	FUBP1(N); USP33(D)	78219349	0.02	0.003	1.15E-10	0.000	0.020	0.86
rs11165643	1	PTBP2(D,N)	96696685	0.022	0.003	2.07E-12	0.030	0.019	0.11
rs543874	1	SEC16B(N)	176156103	0.05	0.004	2.62E-35	0.039	0.019	0.093
rs2820292	1	NAV1(N)	200050910	0.018	0.003	1.83E-10	-0.010	0.020	0.45
rs10182181	2	NCOA1(B)	25003800	0.031	0.003	8.78E-24	-0.020	0.015	0.34
rs1016287	2	LINC01122(N)	59,159,129	0.0229	0.0034	2.25E-11	0.030	0.019	0.17
rs2121279	2	LRP1B(N)	142759755	0.024	0.004	2.31E-08	0.030	0.024	0.18
rs1460676	2	FIGN(N)	164275935	0.021	0.004	4.98E-08	0.020	0.024	0.49
rs1528435	15	UBE2E3(N)	65864222	0.018	0.003	1.20E-08	0.020	0.020	0.21
rs17203016	2	CREB1(B,N); KLF7(B)	207963763	0.021	0.004	3.41E-08	0.020	0.024	0.45
rs7599312	2	ERBB4(D,N)	213121476	0.021	0.003	1.17E-10	0.020	0.015	0.4
rs492400	2	PLCD4(B,Q); CYP27A1(B); USP37(N); TTLL4(M,Q); STK36(B,M); ZNF142(M); RQCD1(Q)	219057996	0.015	0.003	6.78E-09	-0.010	0.015	0.54
rs6804842	3	RARB(B)	25081441	0.018	0.003	2.48E-09	0.020	0.020	0.21
rs2365389	3	FHIT(N	61211502	0.02	0.003	1.63E-10	-0.010	0.015	0.7
rs13078960	3	CADM2(D,N)	85890280	0.029	0.004	1.74E-14	0.020	0.020	0.44
rs16851483	3	RASA2(N)	142758126	0.048	0.008	3.55E-10	-0.010	0.034	0.82
rs13107325	4	SLC39A8(M,N,Q)	103407732	0.047	0.007	1.83E-12	0.039	0.042	0.38
rs11727676	4	HHIP(B,N)	145878514	0.037	0.006	2.55E-08	-0.077	0.045	0.12
rs205262	6	C6orf106(N); SNRPC(Q)	34671142	0.021	0.003	1.75E-10	0.000	0.020	0.97
rs2033529	6	TDRG1(N); LRFN2(D)	40456631	0.018	0.003	1.39E-08	0.020	0.020	0.32
rs2207139	6	TFAP2B(B,N)	50953449	0.045	0.004	4.13E-29	0.039	0.024	0.14
rs9400239	6	FOXO3(B,N); HSS00296402(Q)	109084356	0.017	0.003	1.61E-08	0.010	0.020	0.62
rs13201877	6	IFNGR1(N); OLIG3(G)	137717234	0.024	0.004	4.29E-08	0.030	0.029	0.23
rs13191362	6	PARK2(B,D,N)	162953340	0.029	0.005	7.34E-09	0.020	0.029	0.4
rs1167827	7	HIP1(B,N); PMS2L3(B,Q); PMS2P5(Q); WBSCR16(Q)	75001105	0.02	0.003	6.33E-10	–	0.024	0.24
rs2245368	7	PMS2L11(N)	76,446,079	0.0317	0.0057	3.19E-08	0.049	0.033	0.15
rs6465468	7	ASB4(B,N)	95007450	0.016	0.003	4.98E-08	-0.030	0.019	0.23
rs2033732	8	RALYL(D,N)	85242264	0.018	0.003	4.89E-08	-0.010	0.020	0.63
rs4740619	9	C9orf93(C,M,N)	15624326	0.017	0.003	4.56E-09	0.020	0.020	0.29
rs10968576	9	LINGO2(D,N)	28404339	0.025	0.003	6.61E-14	0.000	0.020	1
rs6477694	9	EPB41L4B(N); C9orf4(D)	110972163	0.017	0.003	2.67E-08	0.010	0.020	0.42
rs1928295	9	TLR4(B,N)	119418304	0.018	0.003	7.91E-10	0.030	0.015	0.12
rs10733682	9	LMX1B(B,N)	128500735	0.019	0.003	1.83E-08	0.030	0.019	0.057
rs7899106	10	GRID1(B,N)	87400884	0.038	0.007	2.96E-08	-0.020	0.034	0.67
rs11030104	11	BDAF(B,M,N)	27641093	0.042	0.004	5.56E-28	0.010	0.025	0.49
rs12286929	11	CADM1(N)	114527614	0.021	0.003	1.31E-12	0.010	0.020	0.5
rs11057405	12	CLIP1(N)	121347850	0.03	0.005	2.02E-08	-0.095	0.044	0.055
rs10132280	14	STXBP6(N)	24998019	0.022	0.003	1.14E-11	0.030	0.019	0.12
rs3736485	15	SCG3(B,D); DMXL2(M,N)	49535902	0.016	0.003	7.41E-09	0.020	0.015	0.29
rs16951275	2	M4P2K5(B,D,N); LBXCOR1(M)	181259207	0.03	0.004	1.91E-17	0.030	0.019	0.21
rs758747	16	NLRC3(N)	3567359	0.023	0.004	7.47E-10	0.000	0.025	0.97
rs3888190	16	ATXN2L(Q); SBK1(Q,D); SULT1A2(Q); TUFM(Q)	28796987	0.031	0.003	3.14E-23	0.010	0.015	0.77
rs1000940	17	RABEP1(N)	5223976	0.018	0.003	1.28E-08	0.010	0.025	0.49
rs1808579	18	NPC1(B,G,M,Q); C18orf8(N,Q)	19358886	0.016	0.003	4.17E-08	0.030	0.019	0.13
rs7239883	18	LOC284260(N); RIT2(B,D)	38401669	0.015	0.003	1.51E-08	0.020	0.015	0.34
rs29941	14	KCTD15(N)	78969207	0.018	0.003	2.41E-08	0.000	0.020	0.92
rs2287019	19	QPCTL(N); GIPR(B,M)	50894012	0.035	0.004	4.59E-18	-0.030	0.029	0.33
rs6091540	20	ZFP64(N)	50521269	0.019	0.003	2.15E-11	0.010	0.020	0.8
rs2836754	21	ETS2(N)	39213610	0.017	0.003	1.61E-08	-0.020	0.020	0.18


### The Influence of BMI on the Risk of T2DM

**FIGURE 3:**
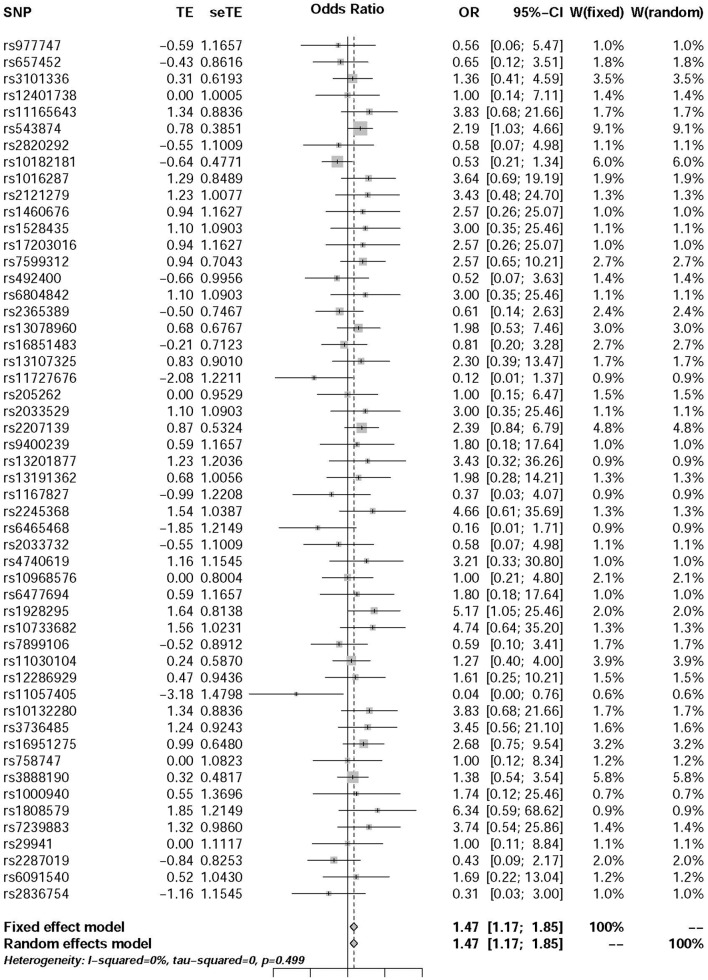
Forest plot of Wald ratios and 95% CIs from BMI-associated SNPs.

The pooled results using IVW method from 52 individual SNPs showed that high BMI significantly increases the risk of T2DM. Due to the lack of evidence of heterogeneity between variants of the summarized data (*P* = 0.499 and *I*^2^ = 0%; Figure 3), the fixed-effect model was utilized here for meta-analysis. The OR of T2DM per *5kg/m^2^* higher BMI was 1.470 (95% CI 1.170 to 1.847; *P* = 0.001). In addition, we analyzed the effect of BMI on the risk of T2DM by six other methods involving Simple median, Weighted median, Penalized weighted median, Penalized IVW, Robust IVW, and Penalized robust IVW methods (Zhao et al., 2017). The results were shown in Table 2, which are consistent with the result based on IVW method.

**Table 2:** Associations of genetic variants with BMI and T2DM.

Method	OR	Lower OR	Upper OR	*P*-value
Simple median	1.767	1.252	2.492	0.001
Weighted median	1.790	1.270	2.524	0.001
Penalized weighted median	1.956	1.383	2.770	0.000
Penalized IVW	1.531	1.215	1.931	0.000
Robust IVW	1.542	1.178	2.016	0.003
Penalized robust IVW	1.573	1.240	1.998	0.000


### Sensitivity Analysis

**FIGURE 4:**
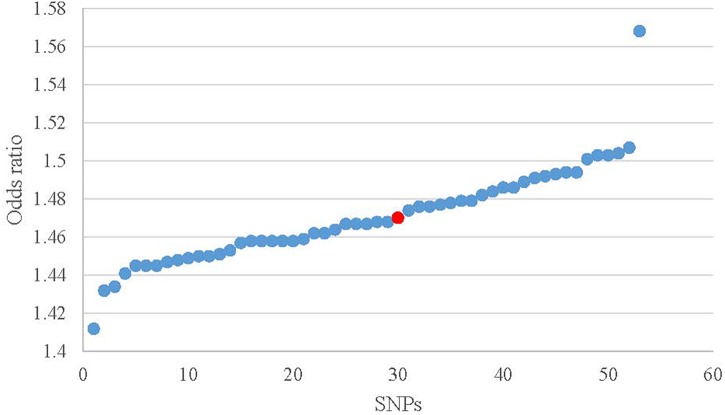
Scatter plot of the ORs in leave-one-out analysis. Red dot is the result without missing SNPs. Blue dots represent the results after missing one SNP.

ORs from leave-one-out analysis were shown in Figure 4. In comparison with the observed result (1.470) from 52 SNPs, the OR increased by 0.075 [(1.568 – 1.470) / 1.470] after removing rs10182181. The ORs after removing other SNPs range from 1.412 to 1.507, which means that the small fluctuation {from -0.039 [(1.412 – 1.470) / 1.470] to 0.025 [(1.507 – 1.470) / 1.470]} can be activated by most of the individual SNPs. These results demonstrated that no single SNP drives the IVW point estimate. The detailed results about Heterogeneity test and meta-analysis of the leave-one-out analysis were shown in the Supplementary Table 2.

### Pleiotropic Effect Analysis

**FIGURE 5:**
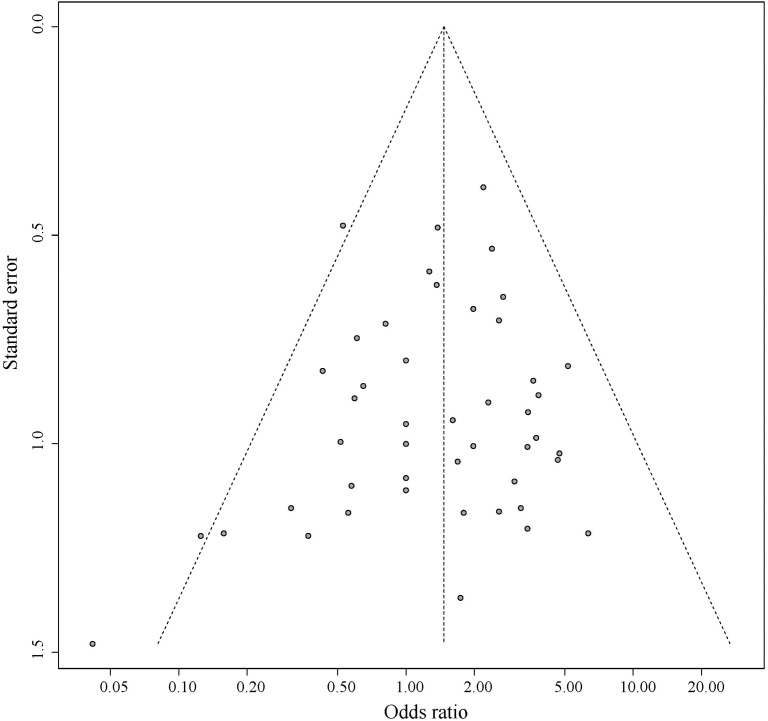
Funnel plot for pleiotropic effect analysis of the variants.

**FIGURE 6:**
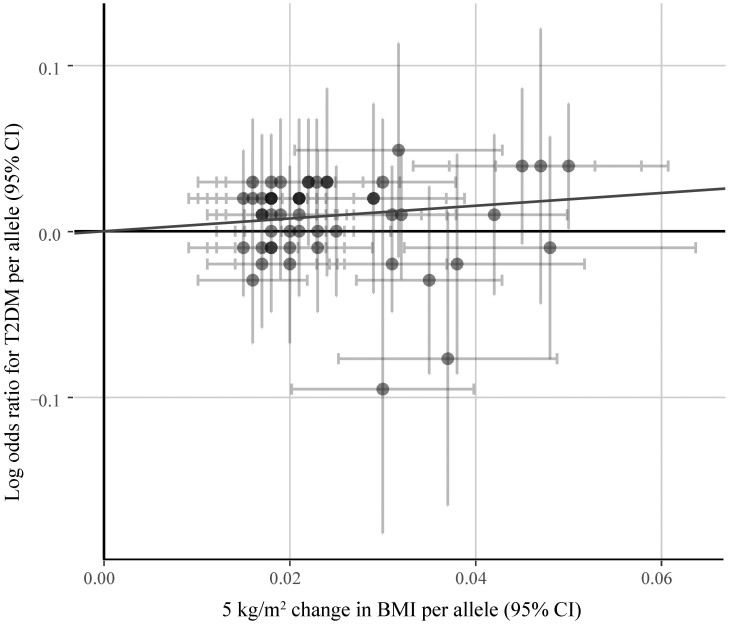
The associations of individual SNPs with BMI and T2DM. Bars represent 95% CIs. The slopes of the blue and black lines show the estimates of genetic variants using IVW method and MR-Egger method, respectively.

Figure 5 shows the symmetrical inverted funnel of the point estimate from individual variants. The effect estimated from MR-Egger regression was 1.24 (95% CI 0.553 to 1.928; *P* = 0.493), with an intercept of 0.004 (95% CI -0.013 to 0.020; *P* = 0.661; Figure 6). Together these findings provided evidence against the possibility that horizontal pleiotropic effects tend to be bias IVW estimates.

## Discussion

In this study, we exposed the causal effect of BMI on the risk of T2DM using MR method. Here, two-summary level data involving association between genetic variants and BMI from Locke et al. (2015) study and association between genetic variants and T2DM from Morris et al. (2012) study were utilized for this purpose. According to the previous investigation, the MR was viewed as a meta-analysis of multiple genetic variants (Bowden et al., 2015; Nordestgaard et al., 2017; Noyce et al., 2017; Wei et al., 2017). Since there was very low heterogeneity between variants of the summarized data (*P* = 0.499 and *I*^2^ = 0%) (Figure 3), the fixed-effect model was utilized for meta-analysis. The pooled results of point estimates using IVW method indicate that the OR of T2DM per *5 kg/m^2^* higher BMI was 1.470 (95% CI 1.170 to 1.847; *P* = 0.001). This evidence suggested that high BMI increases the risk of T2DM.

Sensitivity analysis and bias analysis were then carried out for genetic variants. To test whether the results are influenced by individual SNPs, we conducted the leave-one-out validation. Results in Figure 4 indicate very small fluctuations after the removal of individual SNPs. The statistical evidence of MR-Egger regression (*P* = 0.493) with a very low intercept (0.004; Figure 6) indicates no significant bias of our data and no pleiotropic effect of the genetic variants, respectively.

The inference that the causal effect of BMI on the risk of T2DM from this study is valuable for both investigations and clinical practice. Although abundant observational studies identified the association between BMI and T2DM, a causal effect cannot be ascertained from these investigations. Especially when their common SNPs were identified in recent studies, these genetic variants were then deemed as the primary cause of the BMI-T2DM association by some of the researchers. In brief, current studies cannot help to understand how BMI is associated with T2DM. The observation of this causal effect suggested that helping to decline BMI could be used as a potential method when developing T2DM prevention strategies. Excessive BMI means that the body is overweight or in most cases obese, and this is most likely as the real initial cause of T2DM. Obesity has become a pandemic disease worldwide, which has resulted in a significant increase in the incidence of diabetes, non-alcoholic fatty liver disease and coronary heart disease (Milic et al., 2014; Rao et al., 2015; Zhou et al., 2017). In obesity, the hypertrophy, hypoxia of fat cells, endoplasmic reticulum stress, lipids toxicity and many other factors can lead to adipocytokines dysfunction, increased vascular permeability, along with promoting immune cell infiltration into fat tissue, release of more inflammatory factors, and formation of a vicious circle of inflammatory reactions, leading to the persistence of chronic inflammatory states. It is now widely believed that inflammation plays a key mediator role in the development of type 2 diabetes (Ramalho and Guimaraes, 2008; Engin, 2017). Therefore, strengthening exercise, maintaining a reasonable diet and good fitness are still the iron we must adhere to.

Our study benefits from both the GWAS data and MR method. Clinical statistics using typical methods exposed large number of the associations between diseases and phenotypic exposures. With the rapid increase in the identifications about the genetic basis of diseases and phenotypic exposures, using genetic variants for precise estimates of the causal effect of phenotype on disease by MR method, attracts more and more attention (Benn et al., 2017; Richmond et al., 2017; Rodriguez-Broadbent et al., 2017; Went et al., 2017). For example, Noyce et al. (2017) utilized the MR method for assessing the causal influence of BMI on the risk of Parkinson disease (PD). Nordestgaard et al. (2017) estimated the effect of BMI on Alzheimer’s disease (AD). On account of multiple genetic variants of phenotypes, Bowden et al. (2015) proposed a strategy to view MR with multiple instruments as a meta-analysis and an MR-Egger method for analyzing bias caused by pleiotropy, which was widely used in current studies. Considering the fuzzy relation between BMI and T2DM, we conducted this MR analysis to specify their relationship.

The two assumptions were described in the “Introduction” section for our MR study. Following assumption 1, 97 BMI-associated SNPs were extracted from summary-level data of Locke et al. (2015) study. After removing SNPs with LD and T2DM-associated SNPs, 52 SNPs conforming to the assumption 2 were assigned for further analysis. In addition, MR requires that the genetic variants are independent of any known confounding variables. During to the lack of information about potential confounding factors of BMI and T2DM, no confounders were considered in this study. Therefore, our observation may be limited by this weakness. Link prediction (Liu et al., 2017; Zhang et al., 2017; Peng et al., 2018a) and artificial intelligence methods (Cabarle et al., 2017; Peng et al., 2018b; Wei et al., 2017, 2018b,c) may be used to solve this problem, which has been successfully applied in the prediction of disease genes (Peng et al., 2017; Zeng et al., 2017), miRNAs (Zeng et al., 2016, 2018; Zou et al., 2016), RNA methylation (Wei et al., 2018a), and drug-induced hepatotoxicity (Su et al., 2018).

In summary, the MR analysis in this article verified that high BMI can increase the risk of T2DM. It helps us to understand the pathogenic factor of T2DM. It also may help to enhance the molecular and phenotypic annotations of T2DM and human diseases (Cheng et al., 2016, 2018c), which could be further applied in analyzing diseases in a system biology perspective (Cheng et al., 2018a,b; Hu et al., 2018).

## Author Contributions

LC, JH, RT, and YH conceived and designed the experiments. LC, HZ, HJ, and SY analyzed the data. LC wrote this manuscript. All authors read and approved the final manuscript.

## Conflict of Interest Statement

The authors declare that the research was conducted in the absence of any commercial or financial relationships that could be construed as a potential conflict of interest.

## Footnotes

1 http://cran.r-project.org/web/packages/meta/index.html

## References

[B1] Andreasen C. H. Stender-Petersen K. L. Mogensen M. S. Torekov S. S. Wegner L. Andersen G. (2008). Low physical activity accentuates the effect of the FTO rs9939609 polymorphism on body fat accumulation. *Diabetes Metab. Res. Rev.* 57 95–101. 10.2337/db07-0910 17942823

[B2] Benn M. Nordestgaard B. G. Frikke-Schmidt R. Tybjærg-Hansen A. (2017). Low LDL cholesterol, *PCSK9* and *HMGCR* genetic variation, and risk of Alzheimer’s disease and Parkinson’s disease: Mendelian randomisation study. *BMJ* 357:j3170. 10.1136/bmj.j1648 28438747 PMC5421439

[B3] Bowden J. Smith G. D. Burgess S. (2015). Mendelian randomization with invalid instruments: effect estimation and bias detection through egger regression. *Int. J. Epidemiol.* 44 512–525. 10.1093/ije/dyv080 26050253 PMC4469799

[B4] Cabarle F. G. C. Adorna H. N. Jiang M. Zeng X. (2017). Spiking neural P systems with scheduled synapses. *IEEE Trans. Nanobiosci.* 16 792–801. 10.1109/TNB.2017.2762580 29035221

[B5] Cauchi S. Stutzmann F. Cavalcanti-Proenca C. Durand E. Pouta A. Hartikainen A. L. (2009). Combined effects of MC4R and FTO common genetic variants on obesity in european general populations. *J. Mol. Med.* 87 537–546. 10.1007/s00109-009-0451-6 19255736

[B6] Chen F. Guo Z. Wu M. Zhou Z. Luo W. (2015). [Impact of dynamic changes of waist circumference and body mass index on type 2 diabetes mellitus risk]. *Zhonghua Yu Fang Yi Xue Za Zhi* 49 1092–1097. 26887305

[B7] Chen X. Y. Wu Z. F. Wang X. C. Dong X. L. Zhu J. F. Chen T. (2016). [Association between body mass index and its change and type 2 diabetes mellitus risk in a prospective study]. *Zhonghua Liu Xing Bing Xue Za Zhi* 37 1332–1335. 10.3760/cma.j.issn.0254-6450.2016.10.003 27765120

[B8] Cheng L. Hu Y. Sun J. Zhou M. Jiang Q. (2018a). DincRNA: a comprehensive web-based bioinformatics toolkit for exploring disease associations and ncRNA function. *Bioinformatics* 34 1953–1956. 10.1093/bioinformatics/bty002 29365045

[B9] Cheng L. Jiang Y. Ju H. Sun J. Peng J. Zhou M. (2018b). InfAcrOnt: calculating cross-ontology term similarities using information flow by a random walk. *BMC Genomics* 19:919. 10.1186/s12864-017-4338-6 29363423 PMC5780854

[B10] Cheng L. Wang P. Tian R. Wang S. Guo Q. Luo M. (2018c). LncRNA2Target v2.0: a comprehensive database for target genes of lncRNAs in human and mouse. *Nucleic Acids Res.* 47 D140–D144. 10.1093/nar/gky1051 30380072 PMC6323902

[B11] Cheng L. Sun J. Xu W. Dong L. Hu Y. Zhou M. (2016). Oahg: an integrated resource for annotating human genes with multi-level ontologies. *Sci. Rep.* 6:34820. 10.1038/srep34820 27703231 PMC5050487

[B12] Devuyst O. (2015). The 1000 genomes project: welcome to a new World. *Perit. Dial. Int.* 35 676–677. 10.3747/pdi.2015.00261 26703842 PMC4690620

[B13] Engin A. (2017). The pathogenesis of obesity-associated adipose tissue inflammation. *Adv. Exp. Med. Biol.* 960 221–245. 10.1007/978-3-319-48382-59 28585201

[B14] Frayling T. M. Timpson N. J. Weedon M. N. Zeggini E. Freathy R. M. Lindgren C. M. (2007). A common variant in the FTO gene is associated with body mass index and predisposes to childhood and adult obesity. *Science* 316 889–894. 10.1126/science.1141634 17434869 PMC2646098

[B15] Ganz M. L. Wintfeld N. Li Q. Alas V. Langer J. Hammer M. (2014). The association of body mass index with the risk of type 2 diabetes: a case-control study nested in an electronic health records system in the United States. *Diabetol. Metab. Syndr.* 6:50. 10.1186/1758-5996-6-50 24694251 PMC3976458

[B16] Herder C. Rathmann W. Strassburger K. Finner H. Grallert H. Huth C. (2008). Variants of the PPARG, IGF2BP2, CDKAL1, HHEX, and TCF7L2 genes confer risk of type 2 diabetes independently of BMI in the German KORA studies. *Horm. Metab. Res.* 40 722–726. 10.1055/s-2008-1078730 18597214

[B17] Hu Y. Zhao T. Zhang N. Zang T. Zhang J. Cheng L. (2018). Identifying diseases-related metabolites using random walk. *BMC Bioinformatics* 19:116. 10.1186/s12859-018-2098-1 29671398 PMC5907145

[B18] Lee S. H. Wray N. R. Goddard M. E. Visscher P. M. (2011). Estimating missing heritability for disease from genome-wide association studies. *Am. J. Hum. Genet.* 88 294–305. 10.1016/j.ajhg.2011.02.002 21376301 PMC3059431

[B19] Legry V. Cottel D. Ferrieres J. Arveiler D. Andrieux N. Bingham A. (2009). Effect of an FTO polymorphism on fat mass, obesity, and type 2 diabetes mellitus in the french monica study. *Metabolism* 58 971–975. 10.1016/j.metabol.2009.02.019 19375760

[B20] Liu Y. Zeng X. He Z. Zou Q. (2017). Inferring microRNA-disease associations by random walk on a heterogeneous network with multiple data sources. *IEEE-ACM Trans. Comput. Biol. Bioinform.* 14 905–915. 27076459 10.1109/TCBB.2016.2550432

[B21] Locke A. E. Kahali B. Berndt S. I. Justice A. E. Pers T. H. Day F. R. (2015). Genetic studies of body mass index yield new insights for obesity biology. *Nature* 518 197–206. 10.1038/nature14177 25673413 PMC4382211

[B22] Milic S. Lulic D. Stimac D. (2014). Non-alcoholic fatty liver disease and obesity: biochemical, metabolic and clinical presentations. *World J. Gastroenterol.* 20 9330–9337. 10.3748/wjg.v20.i28.9330 25071327 PMC4110564

[B23] Morris A. P. Voight B. F. Teslovich T. M. Ferreira T. Segre A. V. Steinthorsdottir V. (2012). Large-scale association analysis provides insights into the genetic architecture and pathophysiology of type 2 diabetes. *Nat. Genet.* 44 981–990. 10.1038/ng.2383 22885922 PMC3442244

[B24] Nordestgaard L. T. Tybjaerg-Hansen A. Nordestgaard B. G. Frikke-Schmidt R. (2017). Body mass index and risk of Alzheimer disease: a mendelian randomization study of 399,536 individuals. *J. Clin. Endocrinol. Metab.* 102 2310–2320. 10.1210/jc.2017-00195 28609829 PMC5505195

[B25] Noyce A. J. Kia D. A. Hemani G. Nicolas A. Price T. R. De Pablo-Fernandez E. (2017). Estimating the causal influence of body mass index on risk of Parkinson disease: a mendelian randomisation study. *PLoS Med.* 14:e1002314. 10.1371/journal.pmed.1002314 28609445 PMC5469450

[B26] Olokoba A. B. Obateru O. A. Olokoba L. B. (2012). Type 2 diabetes mellitus: a review of current trends. *Oman Med. J.* 27 269–273. 10.5001/omj.2012.68 23071876 PMC3464757

[B27] Pan A. Sun Q. Manson J. E. Willett W. C. Hu F. B. (2013). Walnut consumption is associated with lower risk of type 2 diabetes in women. *J. Nutr.* 143 512–518. 10.3945/jn.112.172171 23427333 PMC3738245

[B28] Peng J. Hui W. W. Shang X. Q. (2018a). Measuring phenotype-phenotype similarity through the interactome. *BMC Bioinformatics* 19:114. 10.1186/s12859-018-2102-9 29671400 PMC5907215

[B29] Peng J. Zhang X. Hui W. Lu J. Li Q. Liu S. (2018b). Improving the measurement of semantic similarity by combining gene ontology and co-functional network: a random walk based approach. *BMC Syst. Biol.* 12:18. 10.1186/s12918-018-0539-0 29560823 PMC5861498

[B30] Peng J. J. Xue H. S. Shao Y. K. Shang X. Q. Wang Y. D. Chen J. (2017). A novel method to measure the semantic similarity of HPO terms. *Int. J. Data Min. Bioinform.* 17 173–188. 10.1504/IJDMB.2017.084268

[B31] Ramalho R. Guimaraes C. (2008). [The role of adipose tissue and macrophages in chronic inflammation associated with obesity: clinical implications]. *Acta Med. Port.* 21 489–496. 19187692

[B32] Rao W. S. Shan C. X. Zhang W. Jiang D. Z. Qiu M. (2015). A meta-analysis of short-term outcomes of patients with type 2 diabetes mellitus and BMI < / = 35 kg/m2 undergoing Roux-en-Y gastric bypass. *World J. Surg.* 39 223–230. 10.1007/s00268-014-2751-4 25159119

[B33] Richmond R. C. Timpson N. J. Felix J. F. Palmer T. Gaillard R. McMahon G. (2017). Using genetic variation to explore the causal effect of maternal pregnancy adiposity on future offspring adiposity: a mendelian randomisation study. *PLoS Med.* 14:e1002221. 10.1371/journal.pmed.1002221 28118352 PMC5261553

[B34] Rodriguez-Broadbent H. Law P. J. Sud A. Palin K. Tuupanen S. Gylfe A. (2017). Mendelian randomisation implicates hyperlipidaemia as a risk factor for colorectal cancer. *Int. J. Cancer* 140 2701–2708. 10.1002/ijc.30709 28340513 PMC6135234

[B35] Sanada H. Yokokawa H. Yoneda M. Yatabe J. Sasaki Yatabe M. Williams S. M. (2012). High body mass index is an important risk factor for the development of type 2 diabetes. *Intern. Med.* 51 1821–1826. 10.2169/internalmedicine.51.7410 22821094 PMC3540801

[B36] Shi Y. Hu F. B. (2014). The global implications of diabetes and cancer. *Lancet* 383 1947–1948. 10.1016/S0140-6736(14)60886-2 24910221

[B37] Song Y. You N. C. Hsu Y. H. Howard B. V. Langer R. D. Manson J. A. E. (2012). FTO polymorphisms are associated with obesity but not diabetes risk in postmenopausal women. *Obesity* 16 2472–2480. 10.1038/oby.2008.408 18787525 PMC2732012

[B38] Su R. Wu H. Xu B. Liu X. Wei L. (2018). Developing a multi-dose computational model for drug-induced hepatotoxicity prediction based on toxicogenomics data. *IEEE/ACM Trans. Comput. Biol. Bioinform.* 10.1109/TCBB.2018.2858756 30040651

[B39] Voight B. F. Kang H. M. Ding J. Palmer C. D. Sidore C. Chines P. S. (2012). The metabochip, a custom genotyping array for genetic studies of metabolic, cardiovascular, and anthropometric traits. *PLoS Genet.* 8:e1002793. 10.1371/journal.pgen.1002793 22876189 PMC3410907

[B40] Webster R. J. Warrington N. M. Beilby J. P. Frayling T. M. Palmer L. J. (2010). The longitudinal association of common susceptibility variants for type 2 diabetes and obesity with fasting glucose level and BMI. *BMC Med. Genet.* 11:140. 10.1186/1471-2350-11-140 20929593 PMC2958899

[B41] Wei L. Chen H. Su R. (2018a). M6APred-EL: a sequence-based predictor for identifying N6-methyladenosine sites using ensemble learning. *Mol. Ther. Nucleic Acids* 12 635–644. 10.1016/j.omtn.2018.07.004 30081234 PMC6082921

[B42] Wei L. Ding Y. Su R. Tang J. Zou Q. (2018b). Prediction of human protein subcellular localization using deep learning. *J. Parallel Distrib. Comput.* 117 212–217. 10.2174/1566523218666180913110949 30209998

[B43] Wei L. Zhou C. Chen H. Song J. Su R. (2018c). ACPred-FL: a sequence-based predictor based on effective feature representation to improve the prediction of anti-cancer peptides. *Bioinformatics* 34 4007–4016. 10.1093/bioinformatics/bty451 29868903 PMC6247924

[B44] Wei L. Xing P. Shi G. Ji Z. Zou Q. (2017). Fast prediction of methylation sites using sequence-based feature selection technique. *IEEE/ACM Trans. Comput. Biol. Bioinform.* 10.1109/TCBB.2017.2670558 [Epub ahead of print]. 28222000

[B45] Went M. Sud A. Law P. J. Johnson D. C. Weinhold N. Forsti A. (2017). Assessing the effect of obesity-related traits on multiple myeloma using a Mendelian randomisation approach. *Blood Cancer J.* 7:e573. 10.1038/bcj.2017.48 28622301 PMC5520395

[B46] Xi B. Takeuchi F. Meirhaeghe A. Kato N. Chambers J. C. Morris A. P. (2014). Associations of genetic variants in/near body mass index-associated genes with type 2 diabetes: a systematic meta-analysis. *Clin. Endocrinol.* 81 702–710. 10.1111/cen.12428 24528214 PMC5568704

[B47] Yang J. Benyamin B. McEvoy B. P. Gordon S. Henders A. K. Nyholt D. R. (2010). Common SNPs explain a large proportion of the heritability for human height. *Nat. Genet.* 42 565–569. 10.1038/ng.608 20562875 PMC3232052

[B48] Yavorska O. O. Burgess S. (2017). Mendelian randomization: an R package for performing mendelian randomization analyses using summarized data. *Int. J. Epidemiol.* 46 1734–1739. 10.1093/ije/dyx034 28398548 PMC5510723

[B49] Zeng X. Liao Y. Liu Y. Zou Q. (2017). Prediction and validation of disease genes using hetesim scores. *IEEE-ACM Trans. Comput. Biol. Bioinform.* 14 687–695. 10.1109/TCBB.2016.2520947 26890920

[B50] Zeng X. Zhang X. Zou Q. (2016). Integrative approaches for predicting microRNA function and prioritizing disease-related microRNA using biological interaction networks. *Brief. Bioinform.* 17 193–203. 10.1093/bib/bbv033 26059461

[B51] Zeng X. X. Liu L. Lu L. Y. Zou Q. (2018). Prediction of potential disease-associated microRNAs using structural perturbation method. *Bioinformatics* 34 2425–2432. 10.1093/bioinformatics/bty112 29490018

[B52] Zhang S. Zhang D. Jiang Y. Wu L. Shang H. Liu J. (2015). CLU rs2279590 polymorphism contributes to Alzheimer’s disease susceptibility in Caucasian and Asian populations. *J. Neural Transm.* 122 433–439. 10.1007/s00702-014-1260-9 24947876

[B53] Zhang X. Zou Q. Rodruguez-Paton A. Zeng X. (2017). Meta-path methods for prioritizing candidate disease miRNAs. *IEEE/ACM Trans. Comput. Biol. Bioinform.* 16 283–291. 10.1109/TCBB.2017.2776280 29990255

[B54] Zhao Q. Laukkanen J. A. Li Q. Li G. (2017). Body mass index is associated with type 2 diabetes mellitus in chinese elderly. *Clin. Interv. Aging* 12 745–752. 10.2147/CIA.S130014 28496312 PMC5422331

[B55] Zhou Y. Zhang Y. Shi K. Wang C. (2017). Body mass index and risk of diabetic retinopathy: a meta-analysis and systematic review. *Medicine* 96:e6754. 10.1097/MD.0000000000006754 28562529 PMC5459694

[B56] Zou Q. Li J. Song L. Zeng X. Wang G. (2016). Similarity computation strategies in the microRNA-disease network: a survey. *Brief. Funct. Genomics* 15 55–64. 10.1093/bfgp/elv024 26134276

