# Supplementary material for: Exposing the Causal Effect of Body Mass Index on the Risk of Type 2 Diabetes Mellitus: A Mendelian Randomization Study
Source: Front Genet. 2019 Feb 14;10:94. doi: 10.3389/fgene.2019.00094 (PMC6413727; doi:10.3389/fgene.2019.00094)
Supplement: Supplementary file 2 [file Table_2.doc]

**Supplementary Table 2. Sensitivity analysis with each SNP omitted in IVW point estimate.**

| Missing SNPs | Gene | Heterogeneity test | | | Meta-analysis | | |
| --- | --- | --- | --- | --- | --- | --- | --- |
|  |  | *I^2^* | *P* | OR | LOR | UOR | *P* |
| NA |  | 0.000 | 0.499 | 1.470 | 1.170 | 1.847 | 0.001 |
| rs977747 | TAL1(N)   | *TAL1*(N) | | --- | | 0.000 | 0.487 | 1.484 | 1.180 | 1.867 | 0.001 |
| rs657452 | AGBL4(N) | 0.000 | 0.496 | 1.492 | 1.185 | 1.879 | 0.001 |
| rs3101336 | NEGR1(B,C,D,N) | 0.007 | 0.460 | 1.474 | 1.168 | 1.859 | 0.001 |
| rs12401738 | FUBP1(N); USP33(D) | 0.004 | 0.465 | 1.478 | 1.174 | 1.859 | 0.001 |
| rs11165643 | PTBP2(D,N) | 0.000 | 0.507 | 1.445 | 1.148 | 1.819 | 0.002 |
| rs543874 | SEC16B(N) | 0.000 | 0.506 | 1.412 | 1.111 | 1.794 | 0.005 |
| rs2820292 | NAV1(N) | 0.000 | 0.488 | 1.486 | 1.181 | 1.869 | 0.001 |
| rs10182181 | NCOA1(B) | 0.000 | 0.656 | 1.568 | 1.240 | 1.985 | 0.000 |
| rs1016287 | LINC01122(N) | 0.000 | 0.505 | 1.445 | 1.147 | 1.819 | 0.002 |
| rs2121279 | LRP1B(N) | 0.000 | 0.488 | 1.453 | 1.155 | 1.829 | 0.001 |
| rs1460676 | FIGN(N) | 0.003 | 0.468 | 1.462 | 1.162 | 1.838 | 0.001 |
| rs1528435 | UBE2E3(N) | 0.000 | 0.476 | 1.458 | 1.159 | 1.834 | 0.001 |
| rs17203016 | CREB1(B,N); KLF7(B) | 0.003 | 0.468 | 1.462 | 1.162 | 1.838 | 0.001 |
| rs7599312 | ERBB4(D,N) | 0.000 | 0.485 | 1.447 | 1.148 | 1.824 | 0.002 |
| rs492400 | PLCD4(B,Q); CYP27A1(B); USP37(N); TTLL4(M,Q); STK36(B,M); ZNF142(M); RQCD1(Q) | 0.000 | 0.504 | 1.491 | 1.185 | 1.877 | 0.001 |
| rs6804842 | RARB(B) | 0.000 | 0.476 | 1.458 | 1.159 | 1.834 | 0.001 |
| rs2365389 | FHIT(N | 0.000 | 0.516 | 1.503 | 1.193 | 1.893 | 0.001 |
| rs13078960 | CADM2(D,N) | 0.003 | 0.467 | 1.457 | 1.155 | 1.836 | 0.001 |
| rs16851483 | RASA2(N) | 0.000 | 0.487 | 1.494 | 1.186 | 1.883 | 0.001 |
| rs13107325 | SLC39A8(M,N,Q) | 0.002 | 0.469 | 1.459 | 1.159 | 1.836 | 0.001 |
| rs11727676 | HHIP(B,N) | 0.000 | 0.625 | 1.504 | 1.196 | 1.891 | 0.000 |
| rs205262 | C6orf106(N); SNRPC(Q) | 0.004 | 0.466 | 1.479 | 1.175 | 1.861 | 0.001 |
| rs2033529 | TDRG1(N); LRFN2(D) | 0.000 | 0.476 | 1.458 | 1.159 | 1.834 | 0.001 |
| rs2207139 | TFAP2B(B,N) | 0.000 | 0.494 | 1.434 | 1.135 | 1.812 | 0.002 |
| rs9400239 | FOXO3(B,N); HSS00296402(Q) | 0.007 | 0.460 | 1.467 | 1.166 | 1.845 | 0.001 |
| rs13201877 | IFNGR1(N); OLIG3(G) | 0.000 | 0.479 | 1.458 | 1.159 | 1.834 | 0.001 |
| rs13191362 | PARK2(B,D,N) | 0.005 | 0.463 | 1.464 | 1.164 | 1.842 | 0.001 |
| rs1167827 | HIP1(B,N); PMS2L3(B,Q); PMS2P5(Q); WBSCR16(Q) | 0.000 | 0.510 | 1.489 | 1.184 | 1.872 | 0.001 |
| rs2245368 | PMS2L11(N) | 0.000 | 0.509 | 1.449 | 1.151 | 1.822 | 0.002 |
| rs6465468 | ASB4(B,N) | 0.000 | 0.596 | 1.501 | 1.193 | 1.887 | 0.001 |
| rs2033732 | RALYL(D,N) | 0.000 | 0.488 | 1.486 | 1.181 | 1.869 | 0.001 |
| rs4740619 | C9orf93(C,M,N) | 0.000 | 0.477 | 1.458 | 1.159 | 1.834 | 0.001 |
| rs10968576 | LINGO2(D,N) | 0.002 | 0.468 | 1.482 | 1.177 | 1.867 | 0.001 |
| rs6477694 | EPB41L4B(N); C9orf4(D) | 0.007 | 0.460 | 1.467 | 1.166 | 1.845 | 0.001 |
| rs1928295 | TLR4(B,N) | 0.000 | 0.557 | 1.432 | 1.137 | 1.803 | 0.002 |
| rs10733682 | LMX1B(B,N) | 0.000 | 0.512 | 1.448 | 1.150 | 1.821 | 0.002 |
| rs7899106 | GRID1(B,N) | 0.000 | 0.501 | 1.493 | 1.186 | 1.880 | 0.001 |
| rs11030104 | BDAF(B,M,N) | 0.006 | 0.462 | 1.479 | 1.172 | 1.867 | 0.001 |
| rs12286929 | CADM1(N) | 0.007 | 0.459 | 1.468 | 1.166 | 1.847 | 0.001 |
| rs11057405 | CLIP1(N) | 0.000 | 0.692 | 1.503 | 1.195 | 1.890 | 0.000 |
| rs10132280 | STXBP6(N) | 0.000 | 0.507 | 1.445 | 1.148 | 1.819 | 0.002 |
| rs3736485 | SCG3(B,D); DMXL2(M,N) | 0.000 | 0.494 | 1.450 | 1.152 | 1.825 | 0.002 |
| rs16951275 | M4P2K5(B,D,N); LBXCOR1(M) | 0.000 | 0.494 | 1.441 | 1.143 | 1.817 | 0.002 |
| rs758747 | NLRC3(N) | 0.005 | 0.464 | 1.477 | 1.174 | 1.858 | 0.001 |
| rs3888190 | ATXN2L(Q); SBK1(Q,D); SULT1A2(Q); TUFM(Q) | 0.007 | 0.460 | 1.476 | 1.167 | 1.867 | 0.001 |
| rs1000940 | RABEP1(N) | 0.007 | 0.460 | 1.468 | 1.168 | 1.846 | 0.001 |
| rs1808579 | NPC1(B,G,M,Q); C18orf8(N,Q) | 0.000 | 0.518 | 1.450 | 1.153 | 1.824 | 0.001 |
| rs7239883 | LOC284260(N); RIT2(B,D) | 0.000 | 0.495 | 1.451 | 1.153 | 1.825 | 0.002 |
| rs29941 | KCTD15(N) | 0.005 | 0.464 | 1.476 | 1.174 | 1.857 | 0.001 |
| rs2287019 | QPCTL(N); GIPR(B,M) | 0.000 | 0.550 | 1.507 | 1.197 | 1.898 | 0.000 |
| rs6091540 | ZFP64(N) | 0.007 | 0.460 | 1.467 | 1.166 | 1.846 | 0.001 |
| rs2836754 | ETS2(N) | 0.000 | 0.532 | 1.494 | 1.187 | 1.879 | 0.001 |

# Reference

1. Xi B, Takeuchi F, Meirhaeghe A, Kato N, Chambers JC, Morris AP, Cho YS, Zhang W, Mohlke KL, Kooner JS, Shu XO, Pan H, Tai ES, Pan H, Wu JY, Zhou D, Chandak GR, Consortium D, Consortium A-TD, Consortium SD: Associations of genetic variants in/near body mass index-associated genes with type 2 diabetes: a systematic meta-analysis. Clinical endocrinology 2014;81:702-710

2. Morris AP, Voight BF, Teslovich TM, Ferreira T, Segre AV, Steinthorsdottir V, Strawbridge RJ, Khan H, Grallert H, Mahajan A, Prokopenko I, Kang HM, Dina C, Esko T, Fraser RM, Kanoni S, Kumar A, Lagou V, Langenberg C, Luan J, Lindgren CM, Muller-Nurasyid M, Pechlivanis S, Rayner NW, Scott LJ, Wiltshire S, Yengo L, Kinnunen L, Rossin EJ, Raychaudhuri S, Johnson AD, Dimas AS, Loos RJ, Vedantam S, Chen H, Florez JC, Fox C, Liu CT, Rybin D, Couper DJ, Kao WH, Li M, Cornelis MC, Kraft P, Sun Q, van Dam RM, Stringham HM, Chines PS, Fischer K, Fontanillas P, Holmen OL, Hunt SE, Jackson AU, Kong A, Lawrence R, Meyer J, Perry JR, Platou CG, Potter S, Rehnberg E, Robertson N, Sivapalaratnam S, Stancakova A, Stirrups K, Thorleifsson G, Tikkanen E, Wood AR, Almgren P, Atalay M, Benediktsson R, Bonnycastle LL, Burtt N, Carey J, Charpentier G, Crenshaw AT, Doney AS, Dorkhan M, Edkins S, Emilsson V, Eury E, Forsen T, Gertow K, Gigante B, Grant GB, Groves CJ, Guiducci C, Herder C, Hreidarsson AB, Hui J, James A, Jonsson A, Rathmann W, Klopp N, Kravic J, Krjutskov K, Langford C, Leander K, Lindholm E, Lobbens S, Mannisto S, Mirza G, Muhleisen TW, Musk B, Parkin M, Rallidis L, Saramies J, Sennblad B, Shah S, Sigurethsson G, Silveira A, Steinbach G, Thorand B, Trakalo J, Veglia F, Wennauer R, Winckler W, Zabaneh D, Campbell H, van Duijn C, Uitterlinden AG, Hofman A, Sijbrands E, Abecasis GR, Owen KR, Zeggini E, Trip MD, Forouhi NG, Syvanen AC, Eriksson JG, Peltonen L, Nothen MM, Balkau B, Palmer CN, Lyssenko V, Tuomi T, Isomaa B, Hunter DJ, Qi L, Wellcome Trust Case Control C, Meta-Analyses of G, Insulin-related traits Consortium I, Genetic Investigation of ATC, Asian Genetic Epidemiology Network-Type 2 Diabetes C, South Asian Type 2 Diabetes C, Shuldiner AR, Roden M, Barroso I, Wilsgaard T, Beilby J, Hovingh K, Price JF, Wilson JF, Rauramaa R, Lakka TA, Lind L, Dedoussis G, Njolstad I, Pedersen NL, Khaw KT, Wareham NJ, Keinanen-Kiukaanniemi SM, Saaristo TE, Korpi-Hyovalti E, Saltevo J, Laakso M, Kuusisto J, Metspalu A, Collins FS, Mohlke KL, Bergman RN, Tuomilehto J, Boehm BO, Gieger C, Hveem K, Cauchi S, Froguel P, Baldassarre D, Tremoli E, Humphries SE, Saleheen D, Danesh J, Ingelsson E, Ripatti S, Salomaa V, Erbel R, Jockel KH, Moebus S, Peters A, Illig T, de Faire U, Hamsten A, Morris AD, Donnelly PJ, Frayling TM, Hattersley AT, Boerwinkle E, Melander O, Kathiresan S, Nilsson PM, Deloukas P, Thorsteinsdottir U, Groop LC, Stefansson K, Hu F, Pankow JS, Dupuis J, Meigs JB, Altshuler D, Boehnke M, McCarthy MI, Replication DIG, Meta-analysis C: Large-scale association analysis provides insights into the genetic architecture and pathophysiology of type 2 diabetes. Nature genetics 2012;44:981-990

3. Noyce AJ, Kia DA, Hemani G, Nicolas A, Price TR, De Pablo-Fernandez E, Haycock PC, Lewis PA, Foltynie T, Davey Smith G, International Parkinson Disease Genomics C, Schrag A, Lees AJ, Hardy J, Singleton A, Nalls MA, Pearce N, Lawlor DA, Wood NW: Estimating the causal influence of body mass index on risk of Parkinson disease: A Mendelian randomisation study. PLoS medicine 2017;14:e1002314
